# Galleria mellonella as a superficial model for Malassezia globosa and its treatment

**DOI:** 10.1099/acmi.0.000745.v3

**Published:** 2024-06-13

**Authors:** Maritza Torres, Juliana Diaz-Ortiz, Michael G. Davis, James R. Schwartz, Adriana Marcela Celis Ramírez

**Affiliations:** 1 Grupo de Investigación Celular y Molecular de Microorganismos Patógenos (CeMoP), Biological Science Deparment, Universidad de los Andes, Bogotá, Colombia; 2 The Procter & Gamble Company, Mason, OH, USA

**Keywords:** dandruff, *Galleria mellonella*, *Malassezia globosa*, piroctone olamine, zinc pyrithione

## Abstract

**Introduction.***Malassezia globosa* is a yeast species that belongs to the mycobiota of humans and animals, associated with dermatological disorders, such as dandruff. This is a chronic scalp skin disorder characterized by flaking and itching. Treatments include commercial shampoo with different formulations that contain antifungal activities like zinc pyrithione (ZPT) or piroctone olamine (PO). The effectiveness of these formulations has been evaluated for decades for dandruff symptom relief of volunteers. To date, non-mammalian, *in vivo* methods exist to test formulations of these actives.

**Aim.** To evaluate *in vivo* in *Galleria mellonella* larva, two commercial antifungal shampoos (shampoo with 1 % ZPT and 1.6 % zinc Carbonate and shampoo with 0.5 % PO) against this species.

**Methodology.***G. mellonella* larvae were inoculated with *M. globosa* on abraded cuticular surface. Then, integument cell viability, histological changes, and fungal burden were evaluated.

**Results.** Larvae inoculated with *M. globosa* showed higher lesion melanization and tissue damage. In addition, *M. globosa* population showed to increase over time. Concerning the shampoo’s effectiveness, both formulations significantly reduced * M. globosa* burden and tissue damage.

**Conclusion.***G. mellonella* larvae were allowed to evaluate *M. globosa* superficial infection and antifungal effectiveness. Shampoos with ZPT and PO showed a positive effect on inoculated larvae.

## Data summary

All data is presented in the article and one supplementary table (S1) is available in the online version of this article. Table S1 contains the data from the MTT cell-viability assays and the fungal-burden assays carried out in this study.

## Introduction

Belonging to the phylum Basidiomycota, the yeasts of the genus *Malassezia* are part of the commensal skin microbiota of humans and animals [1,2]. These yeasts are found mainly in areas with high sebum production due to their lipid dependence. This is due to the absence of fatty acid synthase (FAS), an enzymatic complex necessary to synthesize *de novo* fatty acids [3,4]. These yeasts have been associated with different systemic and dermatological entities [3]. *M. globosa* is one of the most frequently isolated species from healthy skin in humans [4]. However, under specific conditions, this yeast species can be associated with a causative agent of pityriasis versicolor, seborrheic dermatitis, dandruff, atopic dermatitis, and folliculitis [3,5]. The pathogenicity of *Malassezia* in this entity is not yet clearly known. However, it seems that the interaction of these yeasts with the host’s immune system contributes to the worsening of pre-existing symptoms [3,5].

One of the most common dermatological disorders associated with *Malassezia* is dandruff, characterized by scaly and itchy erythematous lesions [6,7]. This condition is often related to seborrheic dermatitis (SD) since both pathologies share symptoms, risk factors, and treatments. However, desquamation and inflammation of SD appear in areas other than the scalp (neck, face, and chest) and can be more severe. These disorders are linked to the production of lipases that hydrolyse the sebum present in the skin and lead to the release of unsaturated fatty acids and squalene peroxides that trigger the inflammatory response and increase the irritation of the stratum corneum [3,5, 6]. Around 45 % of the general population [8] suffer from (dandruff/SD). The symptoms of these skin disorders can be uncomfortable and even affect the individual’s social development, reducing their quality of life [3,5, 9].

Superficial *in vitro* models, such as co-culturing *Malassezia* spp. with keratinocytes and monocytes, skin equivalent or three-dimensional reconstructed human epidermis have been used to evaluate *Malassezia*–host interaction. Nevertheless, these models are limited due to the fact that they lack tissue vascularization, which would depict the occurring events better during *Malassezia* colonization and invasion [10]. On the other hand, among the *in vivo* models implemented for *Malassezia* are murine models, guinea pigs, canines, and rabbits [2]. These models have been inoculated directly on the skin surface using either occlusion or skin lesioning [2]. Due to their phylogenetic proximity to humans, these models make it possible to reproduce the infectious process and simulate the immune response of a human host. However, using these models is expensive and requires trained personnel, high generation times, a limited number of replicas, and approval of ethics committees [2]. Even though, these mammalian models are required to better understand the host-immune response of the complex skin environment and the associated activation of an adaptative immune response [11]. Due to this and the high prevalence of dandruff, poor knowledge of the host–pathogen interactions, and the need to evaluate commercial antifungal safety, it is important to explore additional *in vivo* models that allow for the determination of infection mechanisms, the virulence factors and the interaction of *Malassezia* with antifungals during a superficial infection.

In response to these limitations, invertebrate models, such as the *Galleria mellonella* larva, have been implemented to evaluate the virulence of fungal pathogens and recently to study host–*Malassezia* interaction during systemic infection [12]. The reasons to use this model are as follows: (1) it is easy to maintain [13], (2) it does not need to be fed through the period in which the model is used (fifth and sixth stages), (3) its wide temperatures range from 25–37 °C, which allows emulating temperatures of the mammalian organism [4,14], are economical to maintain, (5) their size facilitates their manipulation, (6) it does not require approval from an ethics committee, (7) due to its short life cycle, it allows several replicates to be made with a representative number of individuals [13,14], and (8) it has a conservated innate immune system [14].

Three ways of inoculation (injection, oral, and topical) could be used in *G. mellonella* [15]. Since the larval integument is composed by the cuticula, an external physical barrier, that is analogous, in function, to the stratum corneum of the skin, and an epidermal cell monolayer equivalent to the keratinocyte layer in the mammalian epidermis [16], this model may be suitable as a superficial infection model. Considering topical inoculation, a few studies have been reported, mainly filamentous fungal pathogens like *Aspergillus flavus* [17], dermatophytes such as *Trichophyton rubrum*, *T. tonsurans*, *T. equinum*, *Microsporum canis*, *M. gypseum* [18], and entomopathogens like *Beauveria bassiana* [19] have been tested. *G. mellonella* larvae have also been employed to evaluate the effects of miramistine, a topical antifungal, against *Candida albicans* and *Aspergillus fumigatus* [20].

Nevertheless, this model has not been used to evaluate the host–*Malassezia* interaction during superficial infections. This study aims to assess *in vivo*, in the standardized superficial infection model *Galleria mellonella* larva, two commercial antifungal shampoos against this *M. globosa*. The evaluated shampoo was 1 % zinc pyrithione (ZPT) 1.6 % zinc carbonate shampoo (H and S, Procter, and Gamble), and 0.5 % piroctone olamine (PO) shampoo (H and S, Procter, and Gamble). The active components of these shampoo formulations are ZPT (fungistatic) and PO (fungicidal), which are two of the most employed antifungal actives for commercial shampoo formulations [21]. To achieve this goal, *G. mellonella* larvae were abraded to emulate the loss of the epidermal barrier integrity proposed as an essential factor for dandruff and SD [22]. This abrasion was made based on a previous study in which sandpaper was used for this purpose [23]. After abrasion, larvae were inoculated on the cuticular surface with *M. globosa,* and the fungal burden, integument cell viability, and histological changes were evaluated.

## Methods

### Fungal strain and culture

*Malassezia globosa* CBS 7986 (Westerdijk Fungal Biodiversity Institute, Utrecht, The Netherlands) strain was used for assays in which the infection was required. Previous to the assay, the strain was culture at 33 °C during 120 h in modified Dixon agar (mDixon agar) [36 g l^−1^ mycosel agar (BD, USA), 20 g l^−1^ Oxgall (BD, USA), 36 g l^−1^ malt extract (Oxoid, UK), 2 ml l^−1^ glycerol (Sigma Aldrich, USA), 2 ml l^−1^ oleic acid (Sigma Aldrich, USA), and 10 ml l^−1^ Tween 40 (Sigma Aldrich, USA)]. After this, a straw with a diameter of 0.4 mm was used to cut and delimit the area of the culture lawn to be inoculated.

### Abrasion device

**Fig. 1.**
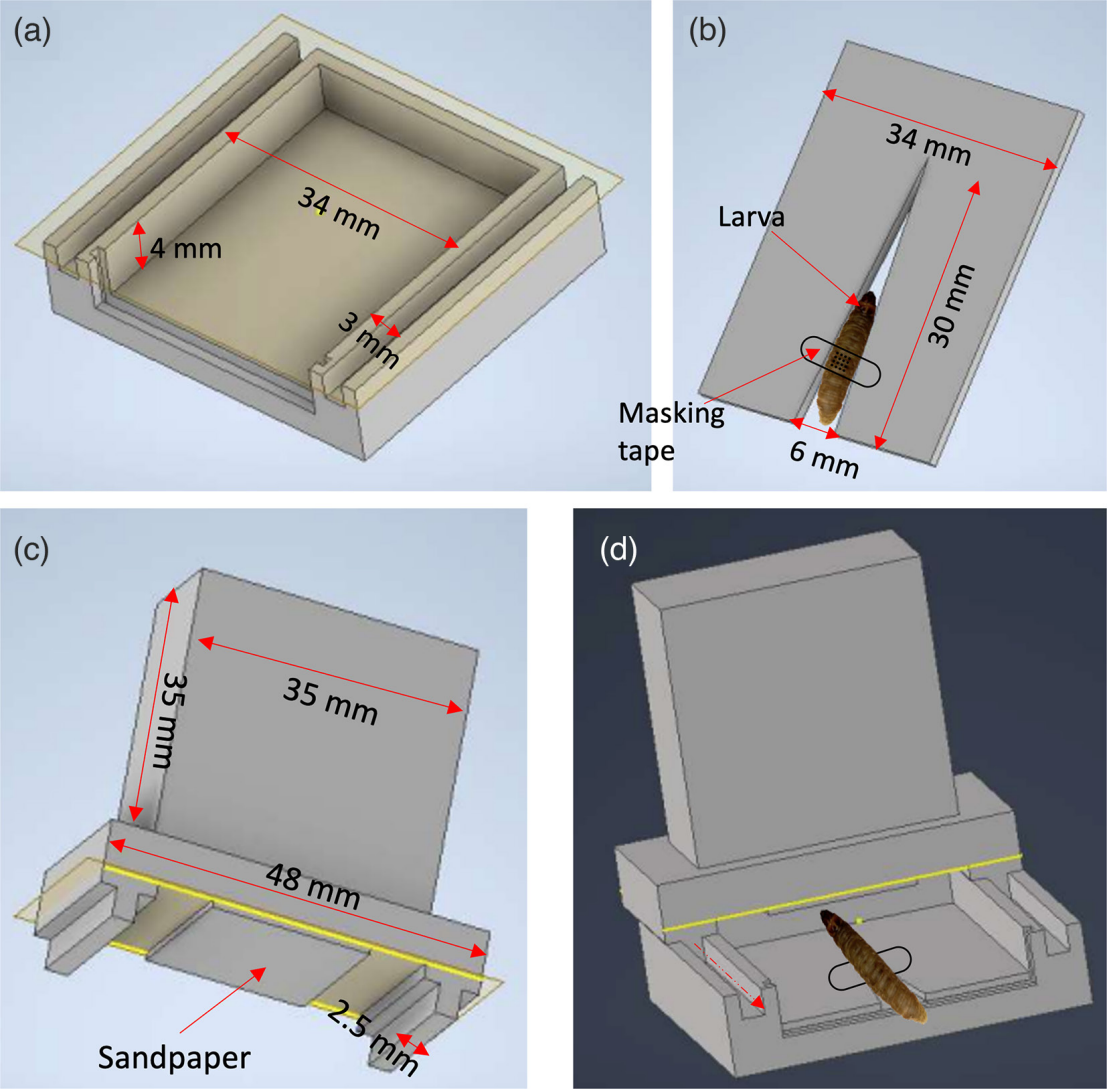
Abrasion device. Dimension and structure of the device with instruction of the assemblage. (**a**) Basal chamber to anchor the abrasion system. (**b**) Acrylic sheet with a notch to restrain the movement of the larva. (**c**) Abrasion system with the sandpaper space. (**d**) Assembled abrasion device (discontinuos line with the red arrow indicates the movement direction of the abrasion system).

The abrasion device was designed based on a previous study [24] with modifications (Fig. 1). This device was constructed using timber to make a chamber (Fig. 1a) that supports a plate of an acrylic sheet of 2 mm as a base (dimensions); on the top of this plate, a piece of acrylic sheet of 2 mm in diameter with a notch of 2.5 mm was placed to restrain the larval movement using masking tape (Fig. 1b). This notch’s size and the acrylic sheet’s diameter allowed abrading larvae from 260 to 280 mg. The timber chamber has two rail channels on each side; in these, the structure with sandpaper (1.5×1.5 cm^2^) (Fig. 1c) joins the chamber, which will allow it to move across the chamber and abrade the larva as the red arrow indicates (Fig. 1d).

### *G. mellonella* larvae pre-treatment

Before each assay, between 260 to 280 mg [25], larvae were weighed, stored in groups of 20 larvae in Petri plates, and incubated at 33 °C. For each assay, larvae were disinfected using a sterile cotton ball impregnated with 70 % ethanol. After this, each larva was placed into the abrasion device using masking tape to prevent larval movement. Each larva was abraded four times. Then, it was placed in a new Petri plate for the corresponding treatment [25].

### Evaluation of the shampoo effect on larval tissue

- **Larval treatment**. Groups of three larvae were put into each of the four treatment groups for this assay. The treatment groups were (1) control larvae (abraded larvae without any shampoo), (2) blank treatment (abraded larvae treated with shampoo without any antifungal active), (3) ZPT treatment (abraded larvae treated with shampoo with 1 % zinc pyrithione, 1.6 % zinc carbonate), and (4) PO treatment (abraded larvae treated with shampoo with 0.5 % piroctone olamine). Larvae belonging to the blank, ZPT, or PO treatments were treated with 3 µl of each of the corresponding shampoos, after 5 min, the shampoo was rinsed out with distilled water, and the larvae were incubated at 33 °C for 1 h or 24 h, each assay was performed in triplicate.
- **Tissue-damage evaluation**. Modifying the tissue evaluation was followed as previously described [26,27] with modifications. Briefly, inoculated larvae and non-inoculated larvae treated with the shampoo formulations were taken to incubation for 5 min at –20 °C, then a cotton swab impregnated with 70 % ethanol was rubbed on the larval surface to eliminate *M. globosa* or any other cellular component that may interfere with the following cell-viability assay. The larvae were dissected to obtain a 0.25 cm^2^ of integument; this piece of tissue was scraped to eliminate tissue different from the integument in the presence of PBS buffer, pH 7.4. The three pieces of the dissected integument of each treatment group were put inside a well of a 48-well plate (Corning Costar) with 200 ml of [3-(4,5-dimethylthiazol-2-yl)-2,5-diphenyl-2H-tetrazolium bromide] (MTT) (Sigma Aldrich, USA) and incubated al 37 °C during 3 h at 100 r.p.m. After this, the pieces of integument were cut for 1 min with a disposable biopsy punch (2 mm diameter) (Medline Industries, USA) in 600 r.p.m. After this, the pieces of integument were cut for 1 min with a disposable biopsy punch (2 mm diameter) (Medline Industries, USA) in 600 ml of isopropanol to dilute formazan crystals and incubated for 2 h at 25 °C at 100 r.p.m. Finally, the 100 ml of each well was taken to a new 96-well flap bottom plate (Corning Costar), and the absorbance was read at 595 nm in a Bio-Rad iMark Microplate Absorbance Reader. The cell-viability percentage was determined as follows:

$$\text{Cell viability}\;(\%)=\frac{OD\;\text{evaluated treatment}}{OD\;\text{control larvae}}\times 100$$

### *In vivo* evaluation of the effectiveness of shampoo against *M. globosa*

- **Larval treatment.** After abrasion, using a 1 ml-inoculating loop, the surface larval abraded area was inoculated with the previously mentioned delimited area of growth of *M. globosa*. Then, the larvae were incubated at 33 °C for 2 h. After this period, according to the treatment group, 3 ml of each shampoo was applied to the larval dorsal surface. Then, after 5 min, the shampoo was rinsed out. The larvae were incubated at 33 °C for 1 h or 24 h. The treatment groups were as follows: (1) control larvae (abraded larvae without either *M. globosa* or shampoo), (2) inoculation group (abraded and inoculated larvae), (3) blank treatment (abraded and inoculated larvae treated with shampoo without any antifungal active), (4) ZPT treatment (abraded and inoculated larvae treated with shampoo with 1 % zinc pyrithione and 1.6 % zinc carbonate), and (5) PO treatment (abraded and inoculated larvae treated with shampoo with 0.5 % piroctone olamine).
- **Integumental fungal burden**. Treated larvae were taken to −20 °C for 5 min. After this period, larvae were euthanized, and the integument of the abraded and inoculated area was put into modified Dixon broth [6 g l^−1^ peptone (Oxoid, UK), 20 g l^−1^ Oxgall (BD, USA), 36 g l^−1^ malt extract (Oxoid, UK), 2 ml l^−1^ glycerol (Sigma Aldrich, USA), 2 ml l^−1^ oleic acid (Sigma Aldrich, USA), and 10 ml l^−1^ Tween 40 (Sigma Aldrich, USA)] during 72 h at 33 °C and continuous agitation at 180 r.p.m. Then, the fragments were plated on mDixon agar and incubated for 15 days at 33 °C, recording the growth of new *M. globosa* colonies daily. Each treatment group was composed of three larvae and were distributed as follows: control larvae (abraded larvae without either *M. globosa* or shampoo), (2) inoculation group (abraded and inoculated larvae), (3) blank treatment (abraded and inoculated larvae treated with shampoo without any antifungal active), (4) ZPT treatment (abraded and inoculated larvae treated with shampoo with 1 % zinc pyrithione and 1.6 % zinc carbonate), and (5) PO treatment (abraded and inoculated larvae treated with shampoo with 0.5 % piroctone olamine). Due to the difficulty of plating and quantifying the number of c.f.u., the growth of *M. globosa* from plated tissue in Dixon agar was scored as one and zero if no growth was evidenced. A recovery percentage was calculated as follows:

 
 
$$\text{Recovery percentage}\;(\%)=\frac{\text{No. of larval tissues with positive yeast recovery}}{\text{No. total larvae evaluated}}\times 100$$

### Histology

Larvae under the different treatments and incubated for 1 h and 24 h were injected with 100 4 % paraformaldehyde in PBS buffer pH 7.4 (PFA). The larvae were kept at 4 °C for 10 days before dehydration in 70 %, 80 %, 90 % ethanol, and absolute isopropanol, followed by paraffin embedding, tissue sectioning each 5 µm, and staining with haematoxylin and eosin (H and E) [12]. The histological images were visualized under a light Leica DM500 microscope.

### Statistical analysis

All experiments were performed in three independent biological replicates; the results from the cell-viability assay and the integumental fungal-burden evaluation were analysed using a two-way ANOVA test. Statistical models were constructed and analysed using GraphPad Prism 8 software (version 8.2.0). A *P*-value of less than 0.05 was considered to be statistically significant.

## Results

### Evaluation of the shampoo effect on larval tissue

**Fig. 2.**
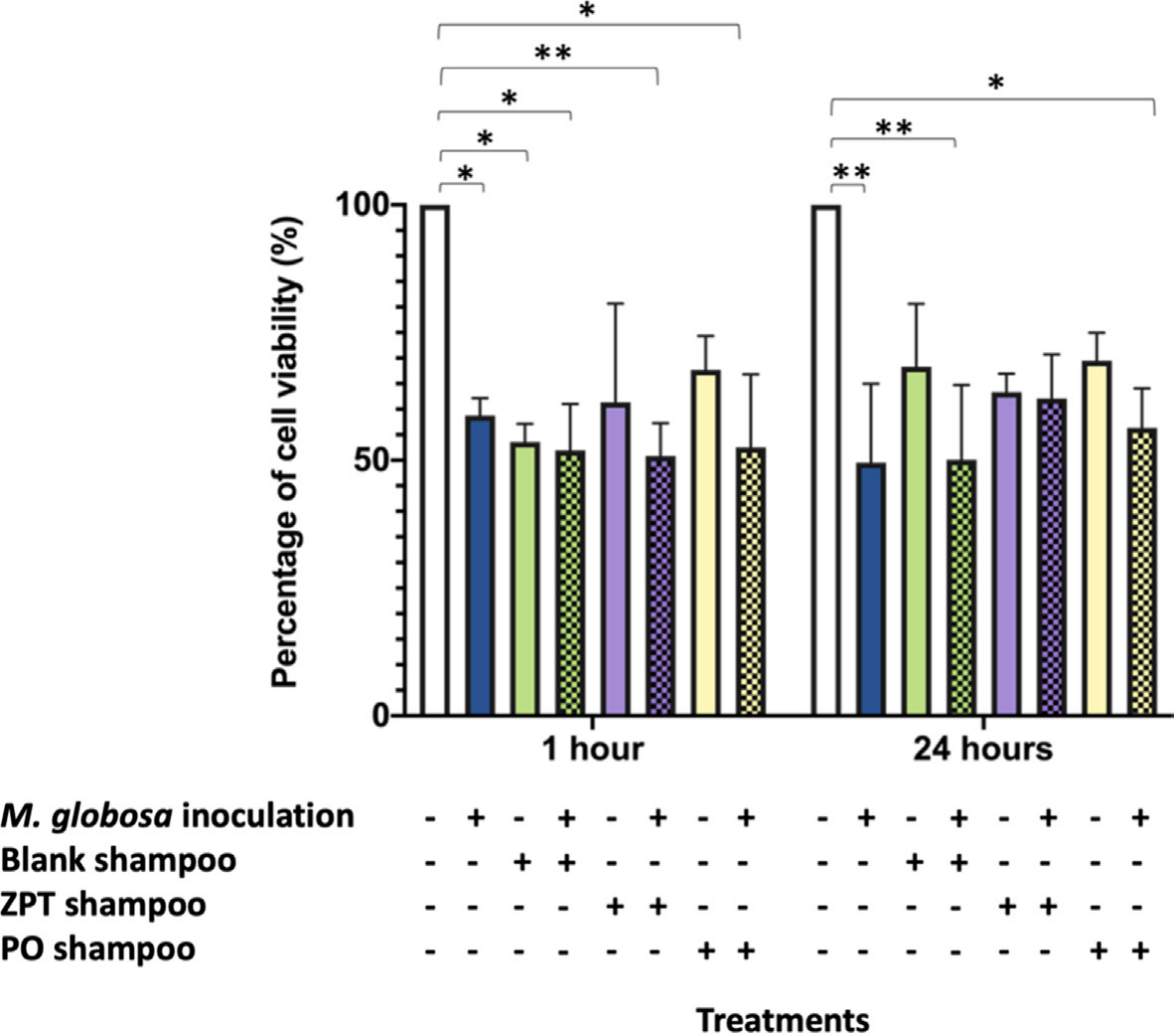
Cell-viability assay of the integument of larvae inoculated with *M. globosa* and treated with an antifungal shampoo. Control larvae were abraded but were not inoculated or treated with antifungal shampoo. Cell viability was evaluated in two different periods. A significant difference was found between treatments (*P*-value=0.0005, two-way ANOVA test). The fill pattern indicates inoculated and treated larvae.

To evaluate the effect of *M. globosa* and the antifungal actives on the integument integrity, an inoculated and treated larval cuticle was dissected, and a cell-viability assay with MTT was performed following the effective time-50 (ET-50) protocol. This protocol determines cell viability equal to or below 50 % at different periods. According to this, substances can be classified as possible corrosive, moderate irritant, moderate to mild irritant, very mild irritant, and non-irritant [26]. In this study, cell-viability assay was performed at two different periods (1) 1 h and (2) 24 h. The results showed that cell viability was significantly different for all treatments (*P*-value=0.0005, two-way ANOVA test), but there was no significant difference for the two periods. At the first hour of evaluation, the integument of larvae inoculated with *M. globosa* and treated with blank shampoo, shampoo with ZPT and PO, and without inoculation and treated with blank shampoo showed to have cell viability significantly lower concerning the control tissue (*P*-value<0.05, two-way ANOVA test). Evaluation at 24 h showed that cell viability of tissue inoculated with * M. globosa* and treated with blank shampoo or PO shampoo was significantly lower than the control tissues (*P*-value<0.05, two-way ANOVA test) (Fig. 2). This decrease was even higher for larvae treated with blank shampoo. Cell viability of integuments during the first hour and after 24 h were not equal or lower than 50 % for the larvae treated with the two antifungal shampoos with the antifungal actives ZPT and PO, meaning that these two shampoos cannot be classified as irritants, as it is indicated in the mentioned study [26] (Fig. 2).

Moreover, in the evaluation at the first hour of treatment, the cell viability of larvae treated with the shampoos with the antifungal actives showed to be higher than the cell viability of larvae treated with the blank shampoo. After 24 h, cell viability for cuticles treated either with the blank shampoo or the shampoos with the antifungal actives showed a slight increase. Otherwise, for larvae inoculated with *M. globosa*, the cell viability decreased after 24 h. A similar effect was observed in larvae inoculated with *M. globosa* and treated with blank shampoo. However, for the time of evaluation and the larvae inoculated with *M. globosa* and not treated with any of the antifungal shampoos or treated with blank shampoo, the integument of larvae treated with the two antifungal shampoos showed an increase in cell viability. This increase was even higher for tissue treated with shampoo with ZPT (Fig. 2).

### *In vivo* evaluation of the effectiveness of shampoo against *M. globosa*

**Fig. 3.**
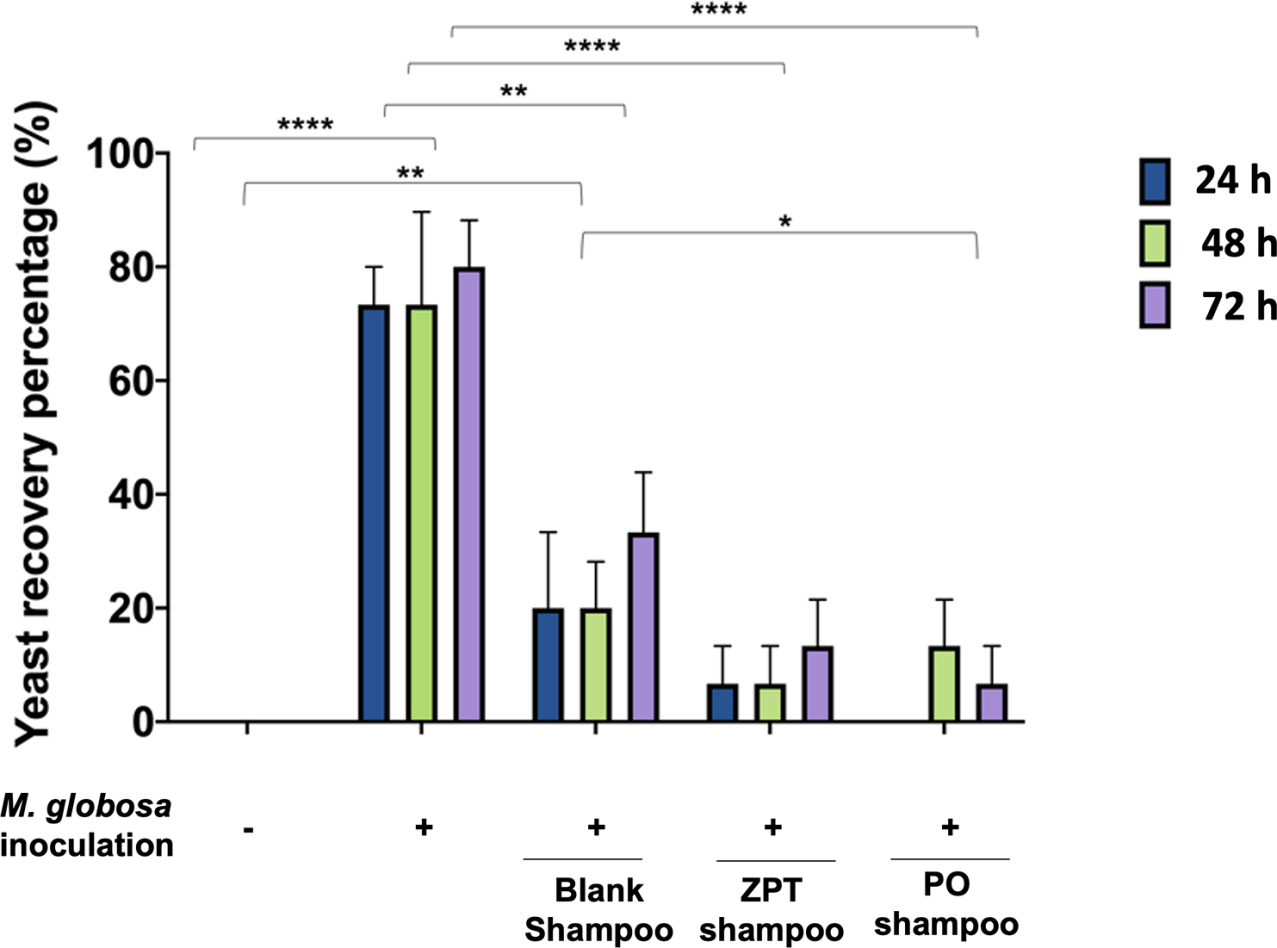
Integumental fungal burden. The percentage of yeast recovery is calculated as previously mentioned. The fungal burden was evaluated in 24, 48, and 72 h periods. A significant difference in fungal burden between treatments was found (*P*-value<0.0001, two-way ANOVA test). There was a higher *M. globosa* recovery from untreated inoculated larvae and a lower yeast recovery from larvae treated with shampoo with PO.

To determine the *in vivo* effectiveness of the two shampoos with the antifungal actives, larvae were inoculated with *M. globosa* and treated with blank shampoo and ZPT or PO. Then, integumental fungal burden was evaluated at 24, 48, and 72 h periods (Fig. 3). The fungal burden evaluation results demonstrated a significant difference (*P*-value<0.0001, two-way ANOVA test) in the percentage of recovery of the yeast of *M. globosa* between treatments. The recovery rate was higher for larvae inoculated with *M. globosa* and without any treatment with shampoo at any of the evaluated periods (24 h, 73 %; 48 h, 73 %; and 72 h, 80 %). This percentage of recovery was followed by the rate of recovery from the larval integument of inoculated larvae and treated with blank shampoo, which reduced the percentages of recovery to 20 % at 24 h, 19.9 % at 48 h, and 33 % at 72 h. The fungal burden of these inoculated tissues and treated with blank shampoo showed a significantly higher recovery percentage concerning control larvae (*P*-value=0.0035, two-way ANOVA test) and a significantly lower recovery percentage in comparison with larvae inoculated with *M. globosa* but without any treatment (*P*-value=0.0019, two-way ANOVA test). Yeast recovery from larvae treated with ZPT or shampoo with PO showed no significant difference. Also, the recovery was not different from the control larvae (*P-*value>0.05, two-way ANOVA test). Likewise, *M. globosa* recovery was significantly lower than from untreated inoculated larvae (*P*-value<0.0001, two-way ANOVA test). Interestingly, the fungal burden tends to increase across time but also tends to remain similar between 24 and 48 h for larvae untreated inoculated larvae, larvae treated with blank shampoo, and shampoo with ZPT. After 72 h, the fungal burden increases for the three experimental groups. Contrary to this, for larvae treated with PO, the fungal burden increases after 48 h and decreases at 72 h (Fig. 3). It is important to mention that *M. globosa* growth was observed only after 10 days of following up the development of c.f.u. from tissue on agar Dixon.

### Histology

**Fig. 4.**
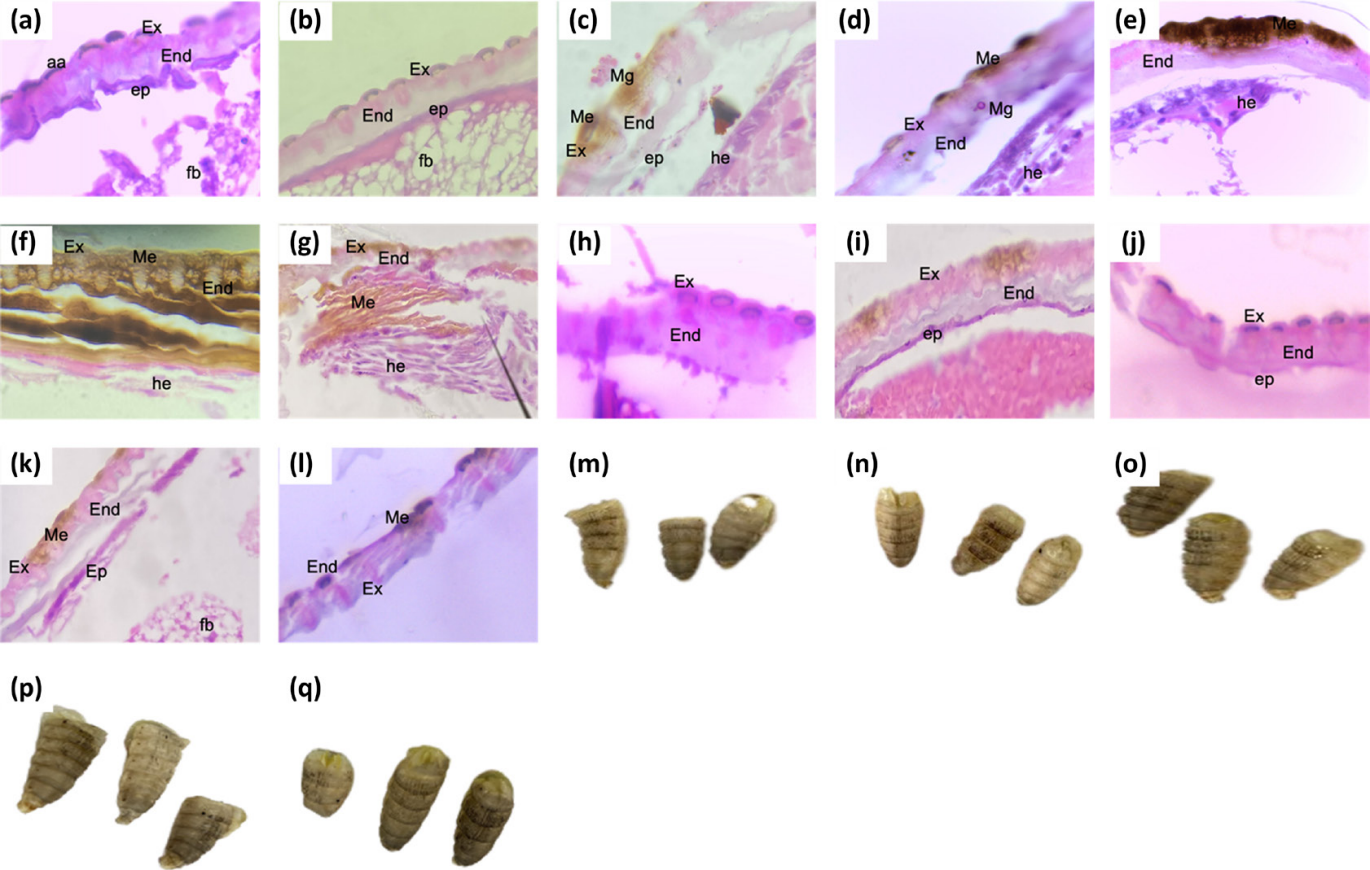
Histological evaluation of integument and macroscopical lesion images. (a)–(l) are histological assessments with H and E of integuments of the following experimental group’s (A and B) abraded control larvae. (**c–f**) Untreated inoculated larvae. (**g**) Larvae treated with blank shampoo. (**h**) Inoculated larvae and treated with blank shampoo. (**i**) Larvae treated with ZPT shampoo. (**j**) Inoculated larvae and treated with ZPT shampoo. (**k**) Larvae treated with PO shampoo. (**l**) Inoculated larvae and treated with PO shampoo. (m)–(q) are macroscopic images of larvae after inoculation and treatment. (**m**) Control larvae. (**n**) Untreated inoculated larvae. (**o**) Inoculated larvae and treated with blank shampoo. (**p**) Inoculated larvae and treated with ZPT shampoo. (**q**) Inoculated larvae and treated with PO shampoo. aa; abraded area, Ex; exocuticle, End; endocuticle; ep; epidermal layer, fb; fat bodies, Me; melanized tissue, Mg; *M. globosa* yeasts, and he; haemocytes.

To understand better the establishment and the effect of the inoculation with *M. globosa* and the treatment with the different shampoo formulations, the cuticle of larvae was fixed and stained with H and E. The tissues were observed under a light microscope to identify possible changes in the tissue composition and the position of *M. globosa* on the cuticle. One of the main changes that could observe after the abrasion is the melanization of the exocuticle in the control larvae (Fig. 4a, b). This dark pigmentation was also observed in larvae under the remaining treatments. However, the melanization was more profound in the tissue affecting the endocuticle, such as in the case of untreated inoculated larvae, larvae treated with blank shampoo, ZPT shampoo, and PO shampoo (Fig. 4c–g, i, k, l) as can be observed in Fig. 4c–f, the tissue in larvae inoculated with *M. globosa* and without any treatment showed higher melanized areas. These areas are consistent with the presence of the yeasts on the surface or in the endocuticle. One of the characteristics of the integument is the presence of a single layer of the epidermal cell, which can be observed in control larvae tissue.

Nonetheless, larvae infected with *M. globosa* present a thicker layer of cells and recruited haemocytes. This haemocyte recruitment can also be observed in larvae treated with blank shampoo (Fig. 4g) and, in part, PO shampoo (Fig. 4k). Concerning tissue integrity, the endocuticle in larvae inoculated with *M. globosa* is affected (Fig. 4f), and the layers of this structure start to separate. A similar change occurs in larvae inoculated with *M. globosa* and treated with either ZPT shampoo (Fig. 4j) or PO shampoo (Fig. 4l); the layers of the endocuticle appeared to lose their integrity but apparently in a lower proportion. Macroscopic evaluation of larvae after 24 h of treatment revealed that inoculated larvae tended to have higher melanization in the abraded area (Fig. 4n) than control larvae (Fig. 4m), *M. globosa*-inoculated larvae and treated with ZPT (Fig. 4p) or PO shampoo (Fig. 4q). Also, inoculated larvae treated with blank shampoo (Fig. 4o) showed darker lesion areas than larvae treated with ZPT shampoo and PO shampoo. However, contrary to inoculated larvae treated with blank shampoo, untreated inoculated larvae presented a reddish scab (Fig. 4n).

## Discussion

The *G. mellonella* larva is an easy-to-handle model implemented as a systemic infection for *M. furfur* and *M. pachydermatis* [12]. In this study, for the first time, *G. mellonella* has been implemented as a superficial infection model for *M. globosa* to evaluate the infection and commercial antifungal shampoo effectiveness. Many studies have implemented diverse *in vitro* protocols to assess the efficacy of different commercial shampoo formulations against *M. furfur, M. restricta,* and *M. globosa* [21,28, 29]. Zinc pyrithione and piroctone olamine have been tested in distinct combinations. Since testing cosmetics on animals has been prohibited in many regions of the globe, *in vivo* evaluation of the effectiveness of anti-dandruff shampoos has relied on human studies to assess dandruff symptom relief [21]. However, assessment of the effect of the interaction of shampoos and *Malassezia* have not been tested *in vivo*, requiring *in vivo* models to evaluate the efficacy of products like anti-dandruff shampoos.

The integument of *G. mellonella* larvae has analogue structures to mammal skin, such as the layer of the cuticle (exocuticle and endocuticle) that has an analogue function with the skin stratum corneous, protecting the tissue from desiccation and epidermal cells monolayer that has a similar role to that of keratinocytes [16]. As one important risk factor for developing dandruff is the loss of skin-barrier integrity [22], in this study, larvae were abraded four times to affect the integrity of the exocuticle and to emulate the condition of skin-barrier disruption. Also, the shampoo formulation was rinsed from the larvae surface as this is the condition in which this kind of product is used. After the treatment with any of the two evaluate shampoos, the larval integument showed an increase cell viability after 24 h, which shows the low irritant potential of the two shampoos being assessed [26].

The final cell viability after 24 h of the integument of larvae inoculated and treated with PO shampoo was significantly lower concerning control larval tissue. Still, the evaluation of the effectiveness of this shampoo showed that it can control the *M. globosa* population on the integument. The cell viability was higher for cuticles treated with ZPT shampoo. That may be because PO has shown a lower adverse effect on *Malassezia* than ZPT when PO is formulated alone without salicylic acid [29]. In contrast, when PO is administrated with salicylic acid [30] or climbazole [21] it has shown a similar or higher effect to that of ZPT against *Malassezia* and the control of dandruff. Also, it has been demonstrated that PO treatments require more extended periods to show a higher positive effect on the scalp of patients with dandruff [21]. The observed low cell viability must be related to the enzymatic activity of lipases, phospholipase, and proteases of *M. globosa*. *In vitro*, treatment with ZPT shampoo (H and S, Procter, and Gamble) has shown that a short exposure time to the shampoo can lead to the reduction of more than 50 % of cell growth of *M. globosa* and almost a 100 % of reduction for *M. restricta* and *M. furfur* [28]. With respect to the blank shampoo, as has been demonstrated previously for non-anti-dandruff shampoo or detergents, these formulations do not inhibit *Malassezia* growth [28]. However, as observed in this study, the effect of enzymatic activity of *M. globosa* and the impact of this non-anti-dandruff shampoo appears to be additive. However, it is expected that with continuous use of these PO and ZPT shampoos, the *M. globosa* population will be lower, so the positive effect of these shampoos will be higher.

Concerning the antifungal effectiveness of both shampoo formulations, ZPT and PO shampoo showed a significant decrease in fungal burden for no treated larvae. After 72 h, the fungal burden star increases for ZPT; however, larvae treated with PO shampoo stars show a decrease in fungal burden after 72 h of incubation. In a previously published study, the substantivity of the antifungal actives PO and ZPT were evaluated, and the results showed that the substantivity of PO was higher, which means that PO could remain longer on the scalp [21], which could be the reason why the shampoo with PO is still inhibiting *Malassezia* growth after 72 h. Due to *Malassezia*’s role in maintaining skin health, it is worth highlighting the importance of maintaining an equilibrium in the yeast population, meaning that treatments should lead to controlling but not eliminating it.

Histological changes in larvae inoculated with *M. globosa* resembled histological changes in the stratum corneous of patients with dandruff; this includes an accumulation of epidermal cells under the lesion area, which make the integument thicker. This epidermal cell migration is part of the wound-healing process of insects [31]. Then, the epidermal cells accumulate under the cuticle, and cell layers detach from it. These alterations in integumental tissue can be reduced using either of the two shampoos’ formulations. An oppositive effect is observed in larvae treated with blank shampoo, in which tissue integrity is lost.

In conclusion, *G. mellonella* larva is a valuable model for studying *Malassezia*–host interaction during superficial interaction. Also, this model can be implemented to evaluate antifungal effectiveness. Here, ZPT and PO shampoos reduced fungal burden and displayed less tissue damage and histological changes associated with *Malassezia* inoculation.

## supplementary material

10.1099/acmi.0.000745.v3 Uncited Supplementary Material 1.

## Abbreviations

FAS: fatty acid synthase
MTT: 3-(4,5-dimethylthiazol-2-yl)-2,5-diphenyl-2H-tetrazolium bromide
PO: piroctone olamine
SD: seborrheic dermatitis
ZPT: zinc pyrithione
